# Monitoring Nasal Breathing Using an Adjustable FBG Sensing Unit

**DOI:** 10.3390/s25134060

**Published:** 2025-06-29

**Authors:** Xiyan Yan, Yan Feng, Min Xu, Hua Zhang

**Affiliations:** 1 Robotics Institute, School of Mechanical and Automotive Engineering, Shanghai University of Engineering Science, Shanghai 201620, China; 15029492776@163.com (X.Y.);; 2 Shanghai Large-Scale Component Intelligent Manufacturing Robot Technology Collaborative Innovation Center, Shanghai 201620, China; 3 School of Information Engineering, Shanghai Zhongqiao Vocational and Technical University, Shanghai 201514, China

**Keywords:** adjustable FBG sensing unit, nasal breathing, breathing frequency, random forest

## Abstract

We have developed an adjustable optical fiber Bragg grating (FBG) sensing unit for monitoring nasal breathing. The FBG sensing unit can accommodate individuals with varying facial dimensions by adjusting the connecting holes of the ear hangers. We employed two FBG configurations: an encapsulated FBG within a silicon tube (FBG_1_) and a bare FBG (FBG_2_). Calibration experiments show the temperature sensitivities of 6.77 pm/°C and 6.18 pm/°C, respectively, as well as the pressure sensitivities of 2.05 pm/N and 1.18 pm/N, respectively. We conducted breathe monitoring tests on male and female volunteers under the resting and the motion states. For the male volunteer, the breathing frequency is 13.48 breaths per minute during the rest state and increases to 23.91 breaths per minute during the motion state. For the female volunteer, the breathing frequency is 14.12 breaths per minute during rest and rises to 24.59 breaths per minute during motion. Experimental results show that the FBG sensing unit can effectively distinguish breathing rate for the same person in different states. In addition, we employed a random forest algorithm to assess the importance of two sensors in breathing monitoring applications. The findings indicate that FBG_1_ outperforms FBG_2_ in monitoring performance, highlighting that pressure plays a positive impact in enhancing the accuracy of breathing monitoring.

## 1. Introduction

In recent years, the importance of monitoring vital signs in daily life has been growing significantly. Among these, breathing parameters serve as critical indicators in the assessment of human health status. The structure monitoring sensor and human compatibility of the sensing unit plays an important role in determining the overall monitoring performance of the breathing equipment. There are various types of sensors for monitoring human breathing, including both non-optical fiber sensors and optical fiber sensors. Non-optical fiber sensors include electrical signal sensors, graphene sensors, effort belts, nasal thermistors, etc. Optical fiber sensors mainly include Fabry-Perot interferometer-based sensors and FBG sensors.

In 2024, Julia et al. designed OptiBreathe—an earable-based PPG system for continuous respiration rate, breathing phase, and tidal volume monitoring [1]. Nora Asyikin Binti et al. have designed a 3D-printed magnetic-based air pressure sensor for continuous respiration monitoring and breathing rehabilitation [2]. Rajendran et al. have designed carbon dots-decorated graphitic carbon nitride as a practical and flexible metal-free nanosensor for breath humidity monitoring [3]. Sharma et al. have designed a crosstalk-free graphene–liquid elastomer based printed sensors for unobtrusive respiratory monitoring [4]. In 2024, Zhang et al. designed a highly humidity-sensitive Fabry-Perot interferometer sensor based on a liquid−solid microcavity for breath monitoring [5]. Shi et al. have designed a flexible wearable fiber-optic sensor for real-time human breath monitoring based on a Fabry-Perot interferometer with an agar film [6]. Kobayakawa et al. have designed a simultaneous measurement of respiratory behavior using three different sensors: pressure transducer-based, belt-type, and thermistor-based methods [7]. The fabrication of the above-mentioned breathing monitoring sensors is complex, and most of the studies evaluated breathing function through exhaled gas humidity measurements. Their functionality is largely determined by the material characteristics, although the structural configuration is comparatively simple.

The optical fiber Bragg grating (FBG) sensor exhibits excellent characteristics such as high linearity, strong anti-interference ability, and ease of implantation, making it suitable for the application of human breathing monitoring [8,9,10].

The FBG sensor can be combined with algorithm models [11], mechanical structures [12,13], biomaterials [14,15], and signal processing [16] to monitor human breathing. These measures optimize the performance of the FBG in breathing monitoring, increase sensitivity, eliminate temperature interference and improve the biocompatibility of the FBG sensor with the human body. In 2023, Liu et al. from Changchun University of Science and Technology fixed an FBG on the chest with an elastic band for the monitoring of breathing and heartbeat frequency [17]. Wang et al.’s research team from Guangdong Province designed a double-layer structure using PDMS (Polydimethylsiloxane), placing an FBG in the middle of the second layer [18]. In 2024, Beijing Normal University developed a wearable micro-fiber intelligent sensor, which uses polymer micro-fiber to monitor human breathing conditions with high efficiency [19]. Based on the reviewed literature, FBG sensors are gaining prominence in breathing monitoring due to their high sensitivity and compact design. Although prior studies have employed FBG sensors, their implementations often necessitate advanced materials with superior mechanical properties and complex signal processing algorithms to achieve reliable data interpretation. Notably, existing research has yet to explore gender-specific variations in breathing patterns—a key innovation addressed in our work.

This work developed an adjustable FBG sensing unit to monitor the breathing rate and temperature, including a bare FBG and a FBG encapsulated within a silicone tube. The schematic diagram of the adjustable FBG sensing unit is described in Section 2. The FBG sensing unit features adjustable connecting holes on the ear hangers, allowing it to suit different facial sizes. Section 3 explains the working principle of nasal breathing monitoring. A normal breathing process exerts pressure on the FBG sensing unit, which undergoes deformation and applies strain to FBGs in proportion to the breathing frequency. In Section 4, the calibration experiments and the nasal breathing experiments under the resting state and the motion state are displayed in detail. In addition, we employed a random forest algorithm to analyze the breathing data. FBG_1_ demonstrates superior monitoring performance compared to FBG_2_, clearly indicating that pressure positively influences nasal breathing monitoring.

## 2. Adjustable FBG Sensing Unit for Monitoring Nasal Breathing

During human breathing, temperature and force variations occur in the nasal cavity. Exhalation exerts an outward thrust, expelling high-temperature air from the body, while inhalation does not produce external force or high temperatures.

To precisely monitor respiratory activity, FBG_1_ is encapsulated in a silicone tube. Since the silicone tube is prone to deformation when subjected to force, the force sensitivity of FBG_1_ is greater than that of FBG_2_.

**Figure 1:**
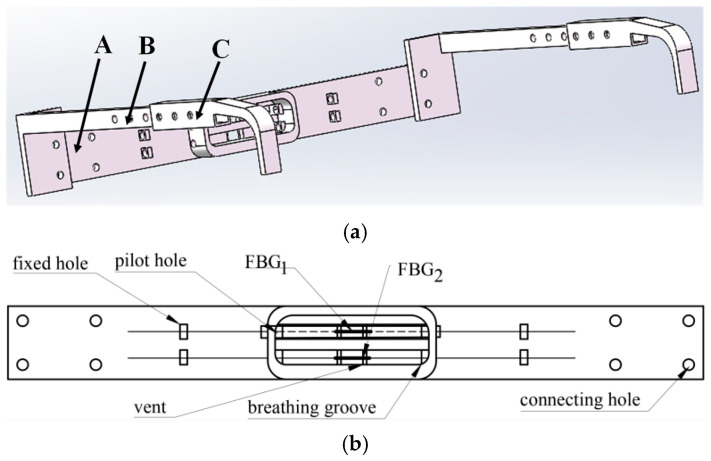

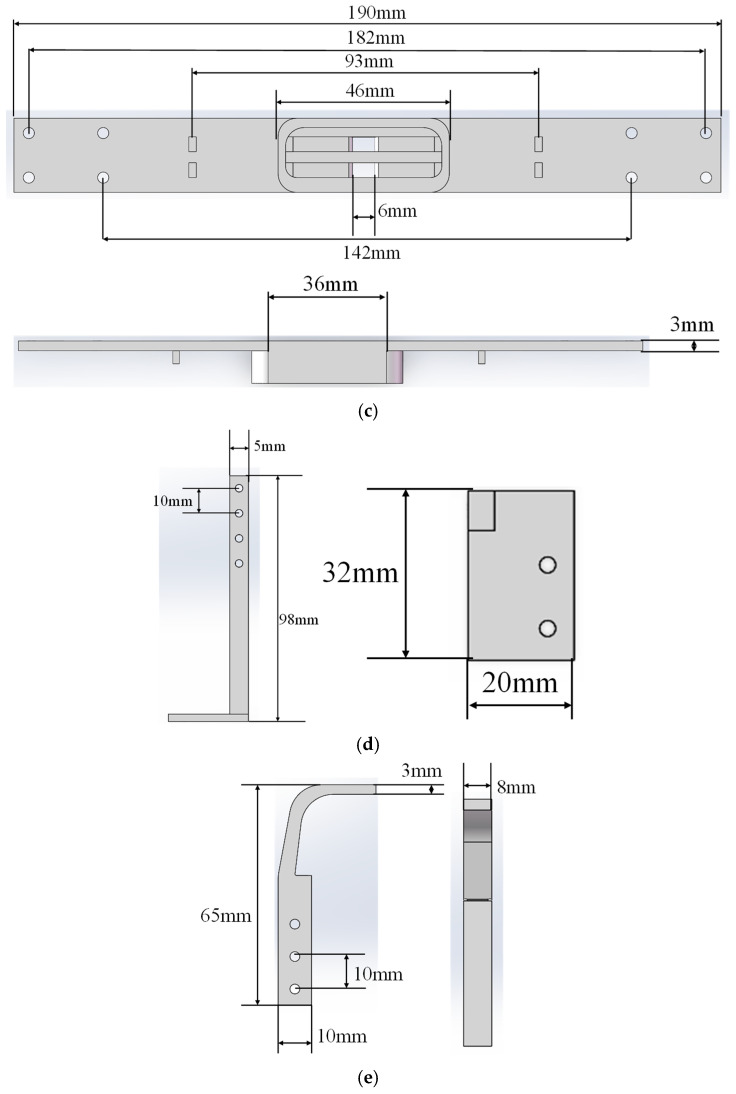
Schematic of the FBG sensing unit: (**a**) assembly drawing; (**b**) part A; (**c**) key dimensions for part A; (**d**) key dimensions for part B; (**e**) key dimensions for part C.

The thermal expansion coefficient of the silicone tube is approximately 200 × 10^−6^/°C, while that of the bare fiber FBG is about 0.55 × 10^−6^/°C. Under the same temperature, the expansion of the silicone tube is greater than that of the bare FBG. The expansion of the silicone tube causes FBG_1_ to stretch, increasing its wavelength. As a result, the temperature sensitivity of FBG_1_ is higher than that of FBG_2_. The details are shown in Figure 1.

The mechanical structure of the monitoring sensing unit was accomplished with 3D printing techniques using a material called PLA (Polylactic acid). Its density typically ranges from 1.24 to 1.30 g/cm^3^, and PLA has a low thermal conductivity. The vent can dissipate the heat during breathing, reducing the temperature effects of FBGs. Part A is the front panel of the FBG sensing unit, used for monitoring breathing status. Part B is the connecting link used to connect part A and part B, and to adjust the distance between the monitoring sensing unit and the nostril. Part C is the hanging leg. It is connected to the ear, like the frame of glasses, and adjusts the distance between the monitoring sensing unit and the nostril. There are connecting holes in part B and part C, allowing people with different face sizes to wear it.

**Figure 2:**
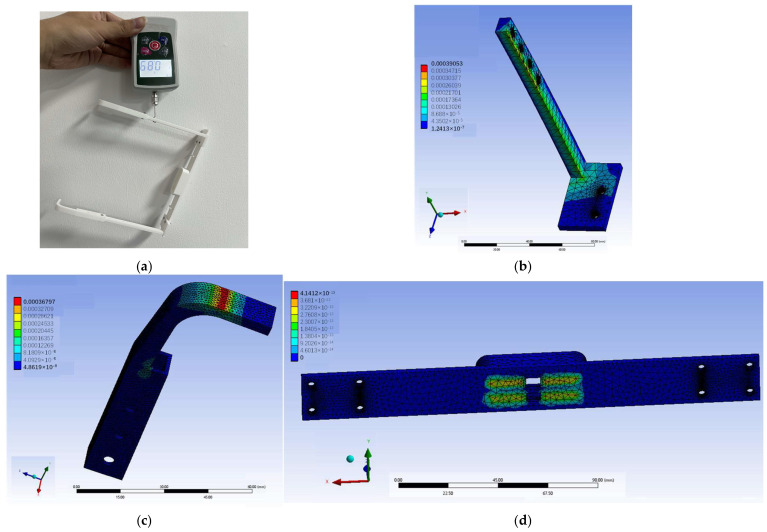
Deformation simulation experiments: (**a**) weighing experiment; (**b**) simulation of part B; (**c**) simulation of part C; (**d**) simulation of part A.

Since the maximum force applied during the force calibration of the sensor in part A was 0.5 N, a 0.5 N load was consequently used for the mechanical simulation of part A. To verify whether the structures of part B and part C could support their own weight, a tensile testing machine (ZMF-50) was used to measure their weight, confirming that it did not exceed 1 N. ANSYS (2022 R1) was then employed to conduct mechanical simulations on part B and part C by applying a 1 N force, determining whether significant deformation occurred, as shown in Figure 2.

The simulation results indicate that part A exhibited no significant deformation under a 0.5 N load, demonstrating the structural integrity of the design. The simulation results demonstrate that neither part B nor part C exhibited significant deformation under a 1 N load, confirming the fundamental structural reliability.

**Figure 3:**
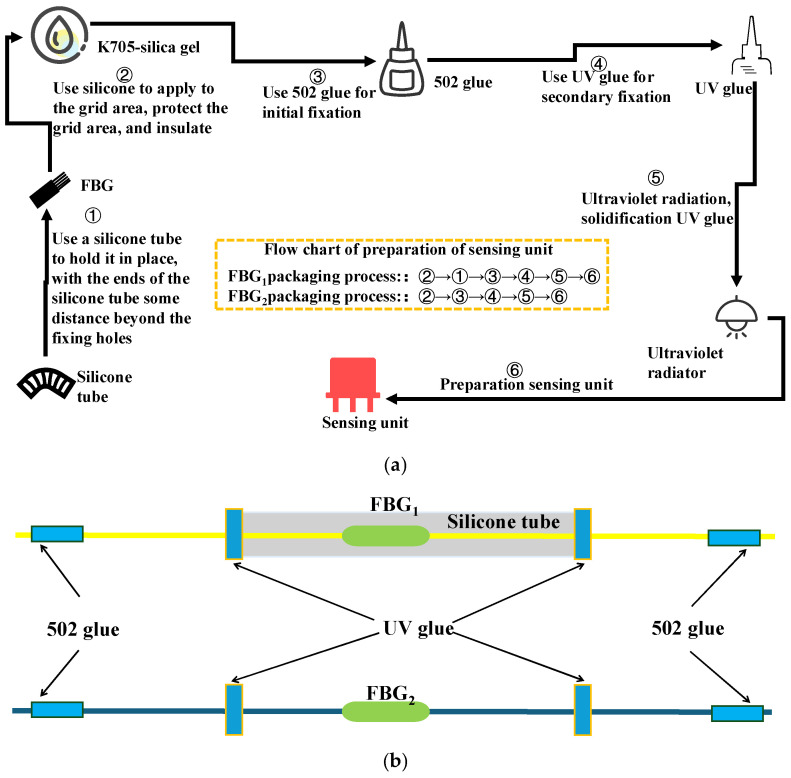
The packaging of the FBG sensor: (**a**) process flow; (**b**) packaged type.

FBG_1_ is capsulated by a silicone tube, and FBG_2_ is bare. The inner diameter of the silicone tube is 0.5 mm. The diameters of the fixed hole and the pilot hole are 3 mm. FBG_1_ uses K-705 silicone to protect its grid area and silicone tubing to encapsulate and protect it, after which it is structurally fixed to the monitoring sensing unit using 502 glue and UV glue, respectively. FBG_2_ uses K-705 silicone to protect its grid area, after which it is structurally fixed to the monitoring sensing unit using 502 glue and UV glue, respectively. The details are shown in Figure 3.

## 3. Working Principle

### 3.1. FBG Sensing Principle

**Figure 4:**
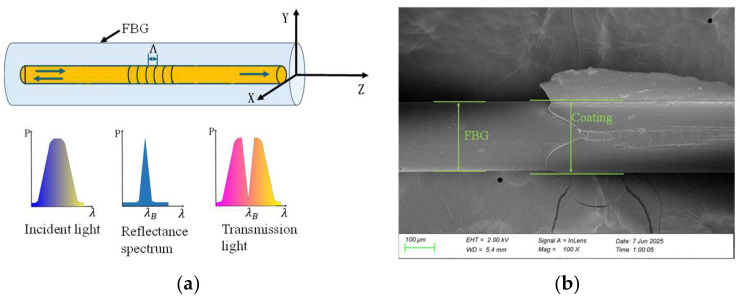

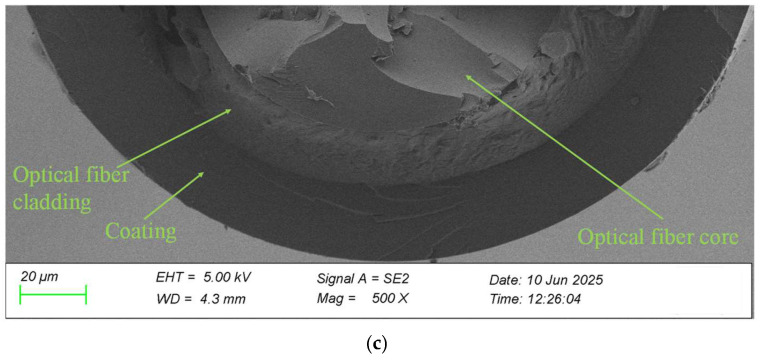
Schematic of the FBG sensor: (**a**) schematic diagram; (**b**) SEM images of the axial view; (**c**) SEM images of the cross-sectional view.

The FBG’s Bragg resonant wavelength $\lambda_{B}$ is the wavelength at which the incident light is reflected by the FBG, expressed as Equation (1) [20,21]. The details are shown in Figure 4.

(1) $$\lambda_{B}=2n_{eff}\Lambda$$

where *n_eff_* is the effective index of the fiber core and Λ is the grating pitch.

FBG’s Bragg resonant wavelength shifts when it is under strain and temperature with $\Delta \lambda_{B}$ expressed as Equation (2)

(2) $$\frac{\Delta \lambda_{B}}{\lambda_{B}}=k_{\varepsilon }\Delta \varepsilon +k_{T}\Delta T$$

where $k_{\varepsilon }$ is the strain sensitivity, $k_{T}$ is the temperature sensitivity, Δε  is the change of axial strain, and ΔT is the temperature change. $P_{e}$ is the effective elastic-optic coefficient, α is the coefficient of thermal expansion, and ξ is the thermal-optical coefficient.

### 3.2. Monitoring Principles

The FBG sensor can sense the strain and temperature simultaneously. Nasal breathing parameters can be reflected via pressure and temperature changes [22,23].

According to Equation (2), the relationships between the wavelengths of FBG_1_ and FBG_2_ and parameters such as strain and temperature can be obtained. Equations (3) and (4) can express these relationships:

(3) $$\frac{\Delta \lambda_{B1}}{\lambda_{B1}}=k_{\varepsilon 1}{\Delta \varepsilon }_{1}+k_{T1}{\Delta T}_{1}$$

(4) $$\frac{\Delta \lambda_{B2}}{\lambda_{B2}}=k_{\varepsilon 1}{\Delta \varepsilon }_{2}+k_{T1}{\Delta T}_{2}$$

Two FBG sensors are used to monitor the breathing rate. The normal breathing process includes exhalation and inhalation, which apply pressure and temperature to the FBG sensing unit. The FBG sensing unit undergoes deformation, thereby causing changes in the wavelengths of FBG_1_ and FBG_2_. The rhythm of the wavelength changes is proportional to the breathing rate. During the breathing process, the wavelength variation rhythms of FBG_1_ and FBG_2_ are consistent, as shown in Equation (5):

(5) $$\Delta \lambda_{B1}/\Delta t=\Delta \lambda_{B2}/\Delta t=k_{f}f$$

where Δt is the time interval during which the strain of FBG increases or decreases by a certain amount during breathing; $k_{f}$ is the breathing coefficient and refers to the frequency coefficient of the volunteers’ breathing, which may vary depending on different individuals and breathing conditions (a higher value suggests an elevated breathing rate within the given time interval, while a lower value corresponds to a reduced breathing rate.); *f* is the breathing rate, which may vary depending on different individuals and breathing conditions (elevated values correspond to increased breathing rates during the measurement period, while diminished values reflect decreased breathing frequency).

## 4. Experiment of Nasal Breathing

It is well known that there is temperature and breathing pressure during nasal breathing. We carried out the temperature calibration and the pressure calibration experiment before the nasal breathing experiment. Four groups of the nasal breathing experiment are listed in Table 1.

### 4.1. Calibration Experiment

**Figure 5:**
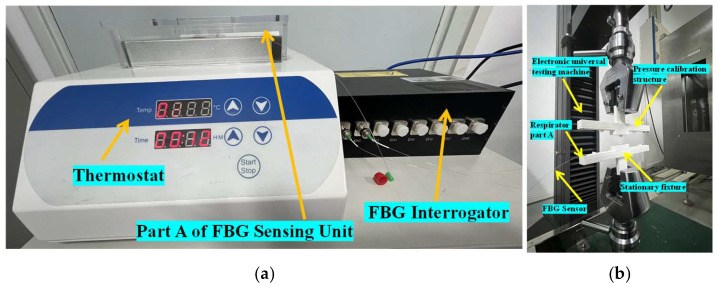
Calibration experimental setup: (**a**) temperature calibration; (**b**) pressure calibration.

Figure 5 shows the calibration experimental setup. Thermostat (GA150-4) (Bin Gu Technology (Shanghai) Co., Ltd., Shanghai, China) was used to control the temperature from 20 °C to 44 °C every 3 °C. The electronic universal testing machine (C65.102) (New Thought (Shanghai) Enterprise Development Co., Ltd., Shanghai, China) was employed to exert loads from 0 N to 0.5 N in increments of 0.1 N. The FBG interrogator (HB-FBG-1000-8) (Huibang (Hangzhou) Sensor Technology Co., Ltd., Hangzhou, China) acquired the Bragg resonant wavelength responses.

**Figure 6:**
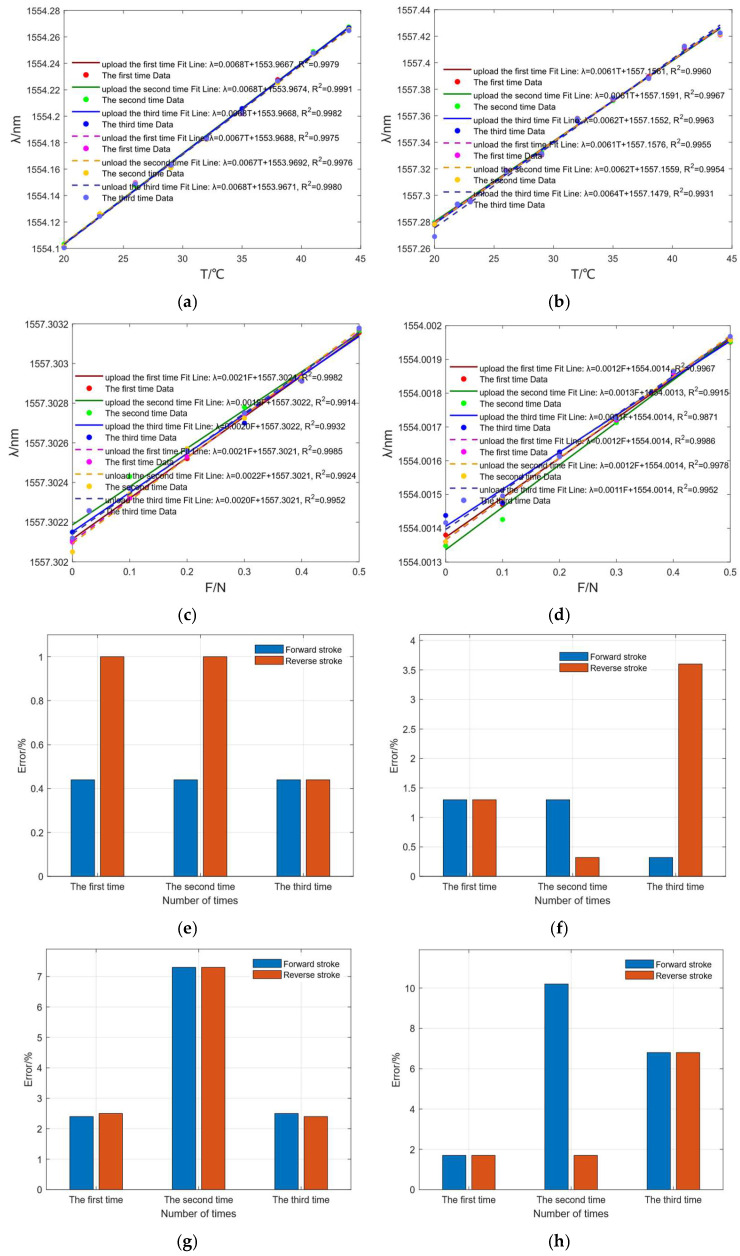
Calibration experiment data: (**a**) FBG_1_’s temperature data; (**b**) FBG_2_’s temperature data; (**c**) FBG_1_’s pressure data; (**d**) FBG_2_’s pressure data; (**e**) FBG_1_’s temperature repeatability error data; (**f**) FBG_2_’s temperature repeatability error data; (**g**) FBG_1_’s pressure repeatability error data; (**h**) FBG_2_’s pressure repeatability error data.

Three calibration experiments were performed for FBG_1_ and FBG_2_ separately, to measure temperature and force during forward and reverse strokes. The repeatability of the sensing unit was evaluated by calculating the error between forward and reverse strokes based on deviations in average temperature and force sensitivity (FBG_1_ and FBG_2_) from their calibration values. The calibration experimental results and error bars are shown in Figure 6.

According to the calibration experiment data, FBG_1_ has an average temperature sensitivity of 6.77 pm/°C and an average goodness of fit of 0.9980, and it has an average pressure sensitivity of 2.05 pm/N and an average goodness of fit of 0.9948. FBG_2_ has an average temperature sensitivity of 6.18 pm/°C and an average goodness of fit of 0.9955, and it has an average pressure sensitivity of 1.18 pm/N and an average goodness of fit of 0.9945. Due to the silicone tube encapsulating and protecting FBG_1_, the silicone tube undergoes significant deformation under stress, resulting in FBG_1_’s force sensitivity being higher than that of FBG_2_. The data in Figure 6e–h indicate that the temperature sensitivity error (compared to the average sensitivity of FBG_1_ and FBG_2_) is consistently below 4% for both forward and reverse strokes under the same conditions. In contrast, the force sensitivity exhibits a higher but still acceptable variation, with a maximum error of around 10%. These findings confirm the high repeatability of the sensing unit in monitoring applications.

**Figure 7:**
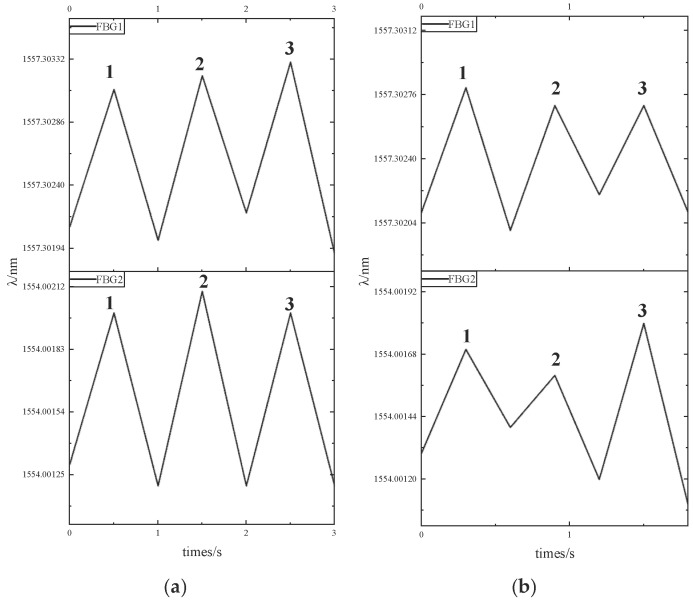
Dynamic calibration experiment data at different force ranges: (**a**) force range of [0 N,0.5 N]; (**b**) force range of [0 N,0.3 N].

To enhance the consistency of force calibration for the sensing units under actual breathing conditions, dynamic pressure calibration experiments were performed. FBG_1_ and FBG_2_ underwent dynamic calibration via an electronic universal testing machine, with cyclic loading ranges of [0 N, 0.5 N] and [0 N, 0.3 N] applied to simulate physiological loading conditions. For each sensor, two separate experimental trials were carried out, with each trial comprising three complete calibration cycles. The dynamic calibration experimental results are shown in Figure 7.

The theoretical wavelength for each cycle peak in the dynamic calibration was determined by multiplying the pressure sensitivity coefficients (obtained from static calibration of FBG_1_ and FBG_2_) by their respective maximum applied forces (the peak forces were 0.5 N and 0.3 N, respectively). The error was then calculated by comparing these theoretical values with the actual peak wavelengths measured during the dynamic calibration experiments. A comparative analysis of the experimental data is presented in Table 2.

Experimental results demonstrate excellent agreement between dynamic and static calibration, with a maximum wavelength deviation of only 0.18 pm between the dynamic calibration peaks and their theoretical counterparts. This close correlation confirms that the monitoring sensing unit maintains consistent performance under both dynamic and static calibration conditions.

### 4.2. Experiments of Group I and Group II

**Figure 8:**
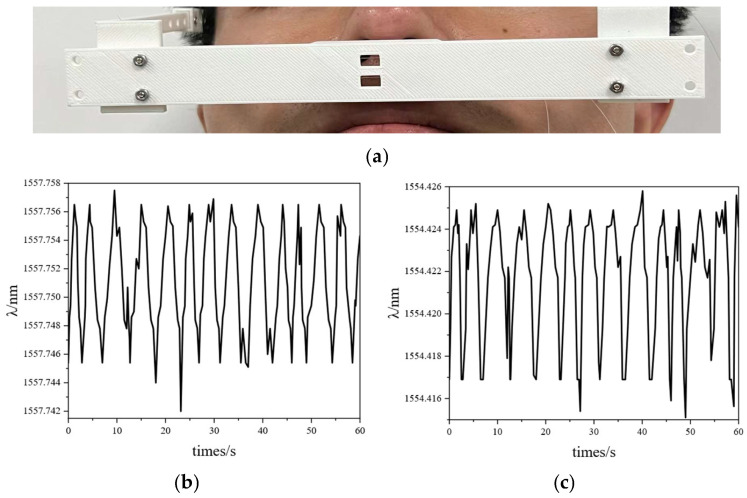

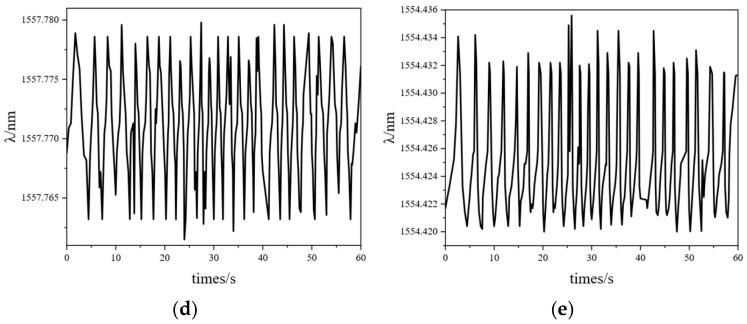
Group I and group II experiments: (**a**) photo of a male’s nasal breathing; (**b**) resting state wave of FBG_1_; (**c**) resting state wave of FBG_2_; (**d**) motion state wave of FBG_1_; (**e**) motion state wave of FBG_2_.

**Figure 9:**
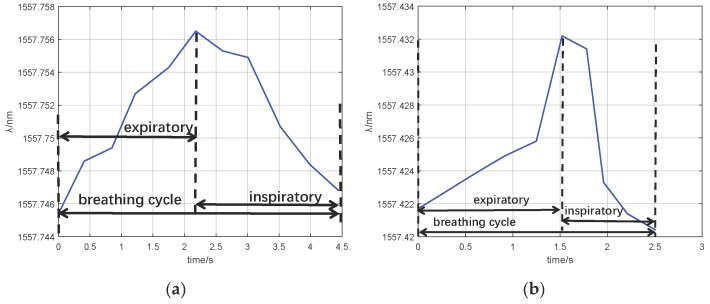
FBG_1_ monitors wavelength changes with one cycle of breathing: (**a**) group I; (**b**) group II.

In the group I experiment, the male volunteer breathed for 60 s. In the group II experiment, he breathed for 60 s after an intense motion (going up and down stairs for 20 min). The FBG interrogator acquired the FBGs’ responses. The experimental data are shown in Figure 8. In addition, Figure 9 illustrates the wavelength shifts during his breathing cycle.

As shown in Figure 8, within 60 s, the breathing rate of the resting male was 13.48 times/min, and that of motion male was 23.91 times/min. The breathing rate of group II was bigger than that of group I. According to Figure 9, the wavelength of expiratory increased while the wavelength of inspiratory decreased. The difference in breathing wavelength in group II was 0.013 nm, and the difference in breathing wavelength in group I was 0. 010 nm. The temperature of group II exhaled was higher than that of group I. As shown in Figure 9, the FBG responses of group I were gentler than that of group II. The breathing rate of group I was lower than that of group II.

### 4.3. Experiments of Group III and Group IV

**Figure 10:**
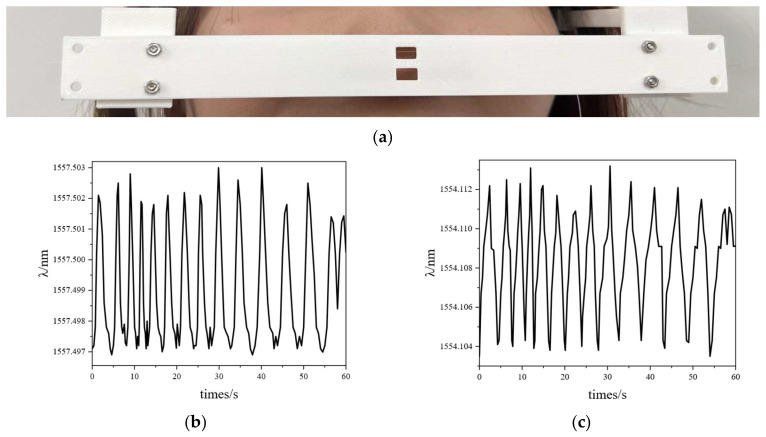

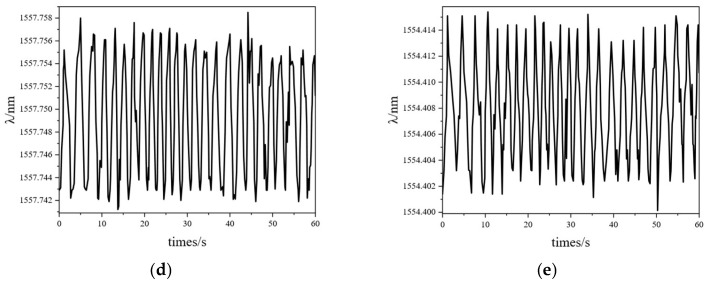
Group III and group IV experiments: (**a**) photo of a female’s nasal breathing; (**b**) resting state wave of FBG_1_; (**c**) resting state wave of FBG_2_; (**d**) motion state wave of FBG_1_; (**e**) motion state wave of FBG_2_.

**Figure 11:**
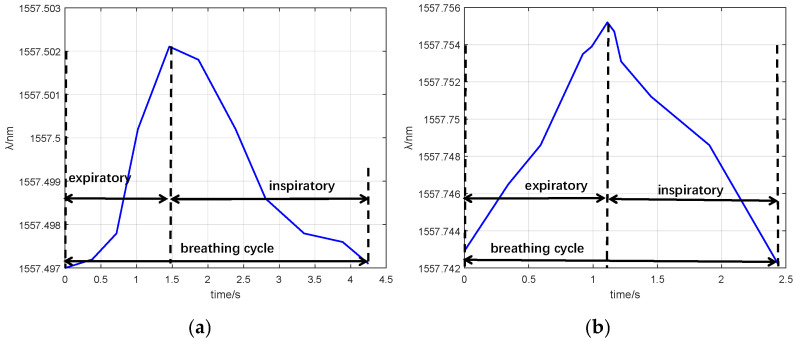
FBG_1_ monitors wavelength changes with one cycle of breathing: (**a**) group III; (**b**) group IV.

In the group III experiment, the female volunteer breathed for 60 s. In the group IV experiment, she breathed for 60 s after an intense motion (going up and down stairs for 20 min). The FBG interrogator acquired the FBGs’ responses. The experimental data are shown in Figure 10. In addition, Figure 11 shows the wavelength shifts during her breathing cycle.

As shown in Figure 10, within 60 s, the breathing rate of resting female was 14.12 times/min, and that of motion female was 24.59 times/min. The breathing rate of group IV was bigger than that of group III. According to Figure 11, the wavelength of expiratory increased, while the wavelength of inspiratory decreased. The difference in breathing wavelength in group IV was 0.011 nm, and the difference in breathing wavelength in group III was 0. 005 nm. The temperature of group IV exhaled was higher than that of group III. As shown in Figure 11, the FBG responses of group IV were gentler than that of group III. The FBG sensing unit could monitor the breathing of the different conditions. The breathing rate of group III was lower than that of group IV.

### 4.4. Verification of the Insignificance of Different Sensors and States

**Figure 12:**
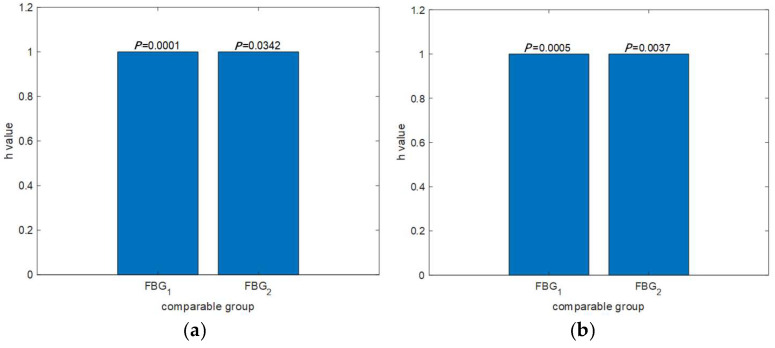
Analysis of the differences between the resting state and the active state: (**a**) group I and group II; (**b**) group III and group IV.

To verify whether the difference between the resting state and the exercise state in the experiment is significant, breathing data from group I, group II, group III, and group IV under different states were analyzed using variance validation. A comparison was conducted using the data monitored from group I, group II, group III, and group IV (FBG_1_ and FBG_2_), as shown in Figure 12.

Through variance analysis, it was found that the variances of FBG_1_ and FBG_2_ in monitoring the data of group I, group II, group III and group IV under different conditions were both below 0.05, indicating that the differences between FBG_1_ and FBG_2_ or between resting and exercise conditions are significant.

### 4.5. Multi-State Verification Experiment

**Figure 13:**
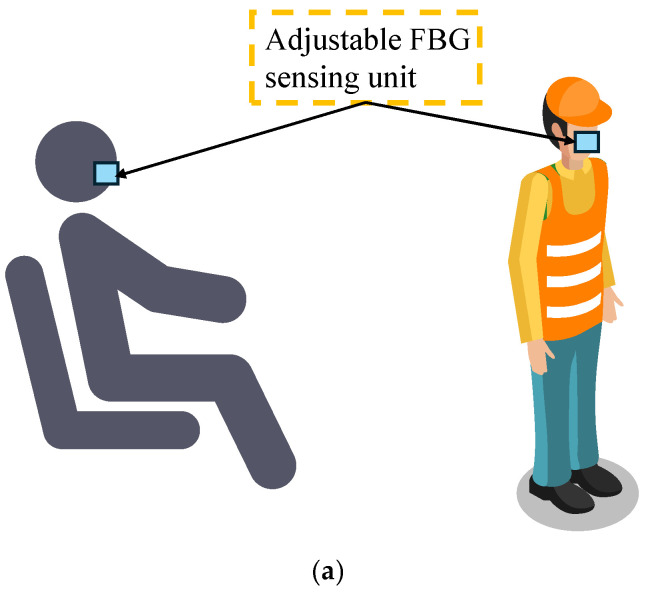

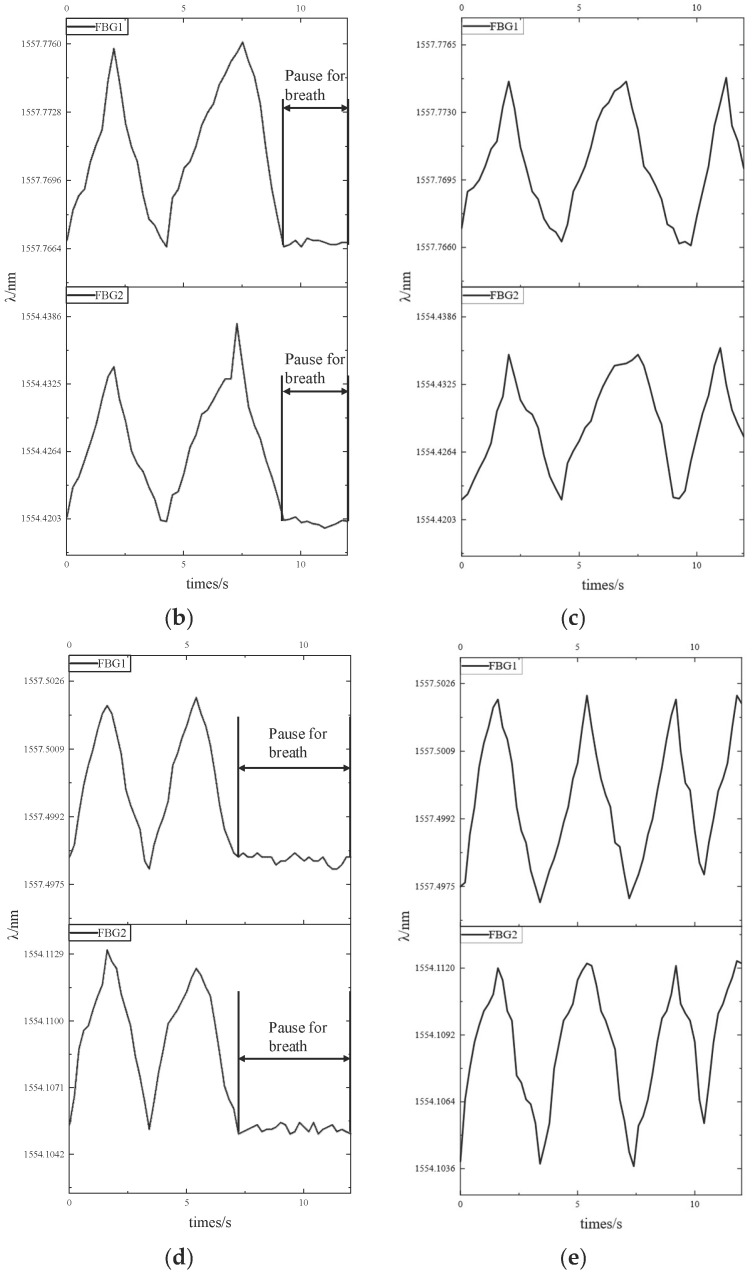
Multi-pose verification experiment: (**a**) monitoring experiment diagram; (**b**) apnea testing stimulated by volunteer 3; (**c**) apnea testing by volunteer 3; (**d**) standing-position monitoring of volunteer 4; (**e**) standing-position monitoring of volunteer 4.

To verify whether the monitoring sensor unit can accurately detect breathing status under different conditions, we designed two experimental scenarios: simulated apnea testing and standing-position monitoring. Preliminary validation was completed using group I, group II, group III, and group IV. To expand the test population, this section’s verification experiments were conducted with two additional subjects: volunteer 3 (a 20-year-old female) and volunteer 4 (a 25-year-old male). The specific experimental setup is shown in Figure 13.

As can be seen in Figure 13, the monitoring sensing unit can correctly differentiate between apnea and breathing conditions, and the condition of breathing while standing can be monitored. This proves that the monitoring sensor unit can monitor more people and has the capability to detect various breathing conditions.

### 4.6. Contrastive Analysis

To verify whether the breathing frequency obtained from the monitoring sensing unit has similarity with the frequency obtained from the same type of sensor and from different types of sensors, breathing monitoring studies from recent years were cited for comparison. The comparative analysis is shown in Table 3.

In normal circumstances (resting), a healthy adult breathes at a rate of 12 to 20 breaths per minute, which agrees with our results [27]. Most current experiments for monitoring breathing do not distinguish between men and women, which is an innovative point for us. The resting and exercising conditions in the above experiments are not identical, and therefore, the breathing rates obtained from monitoring different literature species may vary. The data obtained from the monitoring sensing unit we designed are almost the same as the data obtained from the literature [27], and the trend of the data obtained from other literature is the same (the breathing rate of the exercising state is greater than that of the resting state).

## 5. Experimental Results Analysis

Random forest regression, as a machine learning method, is widely used in medical applications due to its high analyzing accuracy, robustness, and adaptability. The analysis in random forest regression is achieved by constructing multiple decision trees, with each tree trained on a different subset of the training data and the feature set. The final analysis for the target variable is obtained by averaging the analyzes from all the trees, where each tree’s output serves as a “vote” and, in the case of regression, the final analysis is the meaning (or weighted mean) of these outputs.

Based on the above analysis and experimental verification, it can be concluded that the wavelength shift of FBG_1_ during breathing primarily depends on force variations, compared to FBG_2_. In this study, to validate the reliability of the data obtained from the sensor unit and to assess the impact of force on breathing monitoring, we designed a random forest model based on the breathing state to determine which sensor provides the most significant monitoring results.

Random forest was used to analyze the features’ importance. The expiatory phase was defined as 1, and the inspiratory phase was defined as 0. MAE (mean absolute error), MBE (mean deviation error), and RMSE (root mean square error) were calculated in random forest to examine the effect of analyzing features importance:

(6) $$MAE=\frac{\sum_{i=1}^{n}\left|y_{i}-{\hat{y}}_{i}\right|}{n}$$

(7) $$MBE=\frac{\sum_{i=1}^{n}\left|{{\hat{y}}_{i-}y}_{i}\right|}{n}$$

(8) $$RMSE=\sqrt{\frac{\sum_{i=1}^{n}{\left(y_{i}-{\hat{y}}_{i}\right)}^{2}}{n}}$$

where *n* is the number of samples, $y_{i}$ is the true value, and ${\hat{y}}_{i}$ is the analyzed value.

**Figure 14:**
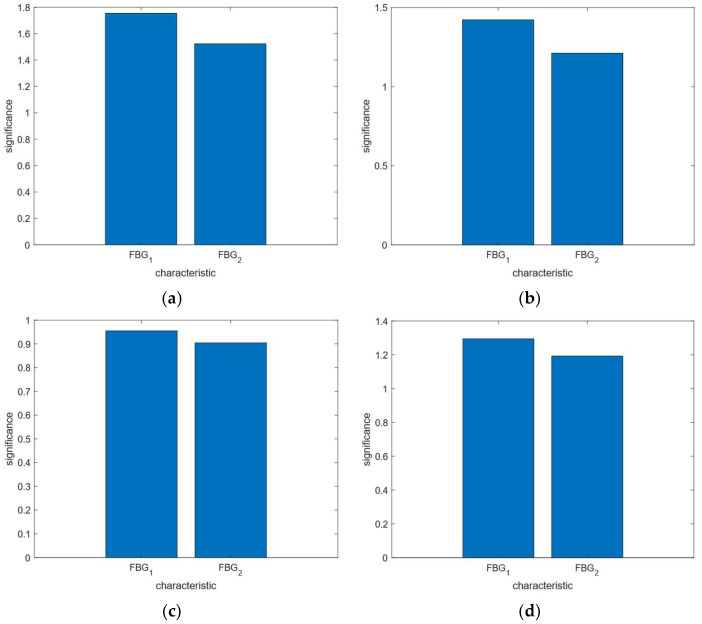
Significance of features in random forest analysis: (**a**) group I; (**b**) group II; (**c**) group III; (**d**) group IV.

In the random forest model used in this paper, the number of decision trees is 250, the minimum leaf size is 30, the depth of the tree is 10, and the method employed is regression. The training set function in the random forest model is TreeBagger, and the prediction set function is predict. The data used for the randomized forest analysis came from the group I, group II, group III, and group IV breathing monitoring experiments. The data of group I are 450, the data of group II are 570, the data of group III are 510, and the data of group IV are 535. For each data set, the training set accounts for 60%, and the test set accounts for 40%. The model parameters were optimized using a five-fold cross-validation method (where evaluation metrics—*MAE*, *MBE*, and *RMSE*—were calculated for each fold and the average of these metrics across all folds was taken as the final overall evaluation metric). The results analyzed are shown in Figure 14.

Table 4 shows the results of *MAE*, *MBE*, and *RMSE*. The maximum difference in *MAE* between Train-set and Test-set is 0.02. The maximum difference in *MBE* between Train-set and Test-set is 0.003. The maximum difference in *RMSE* between Train-set and Test-set is 0.03.

When solving this problem, the random forest model can effectively evaluate feature importance, thereby aiding in feature selection and enhancing the interpretability of the model. By optimizing key hyperparameters, the model’s performance can be further improved. Additionally, it exhibits strong generalization capabilities on unseen data and demonstrates robust resistance to overfitting, making it highly reliable. It can be concluded that the test set of the four groups of data is relatively stable. The monitoring effect of FBG_1_ in the FBG sensing unit is greater than that of FBG_2_, indicating that pressure has a positive impact on breathing monitoring.

## 6. Conclusions

We developed an adjustable FBG sensing unit for monitoring nasal breathing, accommodating individuals with varying facial dimensions. The calibration experiment shows that this FBG sensing unit can monitor the breathing rate and temperature simultaneously. The random forest model analysis indicates the data of nasal breathing detected by the FBG sensing unit are relatively stable. Furthermore, FBG_1_ exhibits a higher monitoring performance than FBG_2_, highlighting that pressure has a positive impact on nasal breathing monitoring.

## Figures and Tables

**Table 1:** Four groups of the nasal breathing experiment.

Group I	Group II	Group III	Group IV
Male’s resting	Male’s motion	Female’s resting	Female’s motion

**Table 2:** Comparison of dynamic calibration peak wavelength and theoretical peak wavelength data.

	Peak Force	Theoretical Wavelength	Actual Wavelengths/Errors at Different Points					
			1	Errors	2	Errors	3	Errors
FBG_1_	0.5 N	1557.30312 nm	1557.3031 nm	0.02 pm	1557.3032 nm	0.08 pm	1557.3033 nm	0.18 pm
	0.3 N	1557.30271 nm	1557.3028 nm	0.09 pm	1557.3027 nm	0.01 pm	1557.3027 nm	0.01 pm
FBG_2_	0.5 N	1554.0019 nm	1554.002 nm	0.1 pm	1554.0021 nm	0.1 pm	1554.002 nm	0.1 pm
	0.3 N	1554.00176 nm	1554.0017 nm	0.06 pm	1554.0016 nm	0.16 pm	1554.0018 nm	0.04 pm

**Table 3:** Comparison of the breathing rates obtained from the monitoring of the proposed sensor with other sensors reported in the literature.

Type	State	Frequency (r/min)	Ref
FBG sensing unit	Male’s resting	13.48	
	Male’s motion	23.91	
	Female’s resting	14.12	
	Female’s motion	24.59	
All-fiber strain-induced humidity sensor	Resting	18	[24]
	Motion	52.8	
Conductive hydrogel	Resting	18	[25]
	Motion	57	
All-nanofiber triboelectric sensor	Resting	about 24	[26]
	Motion	about 36	
Ultra-fast moisture sensor	Resting	about 19.3	[27]
	Motion	about 36.4	
Double-fishtail-shaped FBG	Resting	9.6	[28]
	Motion	20.4	
Interlock-stitched knitted structure	Resting	about 24	[29]
	Motion	about 60	

**Table 4:** Predicting results.

	Group I	Group II	Group III	Group IV
Train-set *MAE*	0.20	0.16	0.21	0.27
Test-set *MAE*	0.21	0.15	0.19	0.28
Train-set *MBE*	0.007	0.008	0.004	0.005
Test-set *MBE*	0.004	0.009	0.007	0.003
Train-set *RMSE*	0.29	0.18	0.22	0.35
Test-set *RMSE*	0.27	0.17	0.19	0.36

## Data Availability

The experimental data are available from Shanghai Large-scale Component Intelligent Manufacturing Robot Technology Collaborative Innovation Center or by contacting the authors directly.
